# The Long Road of Immunotherapeutics against Multiple Sclerosis †

**DOI:** 10.3390/brainsci10050288

**Published:** 2020-05-11

**Authors:** Vasso Apostolopoulos, Abdolmohamad Rostami, John Matsoukas

**Affiliations:** 1 Institute for Health and Sport, Victoria University, Melbourne, VIC 3030, Australia; vasso.apostolopoulos@mail.com; 2 Neurology Department, Thomas Jefferson University, Philadelphia, PA 19107, USA; A.M.Rostami@jefferson.edu; 3 NewDrug, Patras Science Park, 26500 Patras, Greece

**Keywords:** multiple sclerosis, MS, vaccine, immunomodulation, carriers

## Abstract

This commentary highlights novel immunomodulation and vaccine-based research against multiple sclerosis (MS) and reveals the amazing story that triggered this cutting-edge MS research in Greece and worldwide. It further reveals the interest and solid support of some of the world’s leading scientists, including sixteen Nobel Laureates who requested from European leadership to take action in supporting Greece and its universities in the biggest ever financial crisis the country has encountered in the last decades. This support endorsed vaccine-based research on MS, initiated in Greece and Australia, leading to a worldwide network aiming to treat or manage disease outcomes. Initiatives by bright and determined researchers can result in frontiers science. We shed light on a unique story behind great research on MS which is a step forward in our efforts to develop effective treatments for MS.

## 1. Introduction

### Nobel Laureates Taking Action to Support Research in Greece

It was realized and clearly understood by the governments in Greece five years ago, and especially now, during this period of COVID-19 pandemic, the necessity for research, as first priority in their policies, for innovation, development and growth. Greece has suffered a lot the last decade from recession. Initiatives by eminent scientists were taken to support research in Greece, with remarkable positive outcomes. Fifteen Nobel Laureates cosigned the “Support for Greece” petition that was addressed by Nobel Laureate Professor Harald zur Hausen, on 14 December 2015, to the European leadership (Jean-Claude Juncker, Martin Schulz and Donald Tusk), pleading for the support for research in universities and to the country. The first to sign was the DNA discoverer Nobel Laureate Professor James Watson, who also sent a letter to the then President of the USA, Mr. Barack Obama, urging him to support Greece [1]. This petition to support research and universities in Greece led to the Hellenic Foundation for Research and Innovation (HFRI) to spur economic development. The European Investment Bank co-financed the creation of the HFRI fund with the Ministry of Finance. Professor Costas Fotakis, Alternate Minister of Research and Technology then, has greatly contributed to the establishment of HFRI. The HFRI fund launches regular calls for all scientists at all stages in support of their research. The “Support for Greece” petition, which was co-signed by the Nobel Laureates and led to the HFRI fund, was a joint initiative between Professor John Matsoukas from the University of Patras Greece and Nobel Laureate Professor Harald zur Hausen from the German Cancer Research Center in Heidelberg, Germany. This is the second petition after the first in 2012 co-signed by twenty-one Nobel Laureates and addressed again by Professor Harald zur Hausen [2,3]. The second petition worked out successfully.

## 2. The Sparkle of Immunotherapeutics MS Research

Nobel Laureates Professors James Watson (Cold Spring Harbor Laboratory, New York, NY, USA), Harald zur Hausen (German Cancer Research Center, Heidelberg, Germany) and Andrew Schally (University of Miami, FL, USA) were attracted by the excellent research in Greece and have stated in particular that MS research in Greece is world-class research that is worthy of support. This research had its reason and sparkle. Myelin based immunotherapeutics research for MS in Greece was triggered by Dr. Elizabeth Matsoukas, a Biologist, who has been struck by the disease. That happened to her in 1982, at the age of 30. Following her diagnosis, she dedicated her life to promote research for MS. Her PhD dissertation from the National Hellenic Research Foundation in Athens identified and evaluated myelin epitopes, in particular myelin basic protein (MBP) epitopes, which are implicated in the pathogenesis of the disease [4,5,6,7]. Now these epitopes are the tools and the core for developing therapeutics and vaccines for the treatment of MS. 

## 3. The First EAE Experiment in Pennsylvania

In 1994, Professor John Matsoukas, brother of Elizabeth, decided to introduce into his drug discovery research program at the University of Patras the design, synthesis and development of drugs, mimetics and immunotherapeutics, using MBP epitopes against MS. The first experiment, “experimental allergic encephalomyelitis” (EAE), an animal model of the disease, was run that year, at the University of Pennsylvania, in Professor Abdolmohamad Rostami’s lab, at that time professor of neurology at the University of Pennsylvania (currently Professor and Chairman of the Department of Neurology at Thomas Jefferson University, Philadelphia USA) [8,9,10]. Elizabeth had visited Professor Rostami earlier that year in his Pennsylvania clinic for diagnosis and prescription of an interferon drug for her case which was not possible yet at that time in Europe. First experiments were carried out, using the guinea pig epitope MBP_72–85_, as suggested by Elizabeth [10]. The research for new therapies was part of her curiosity to determine the mechanisms of disease and, based on that, to pursue treatment of disease. Her first EAE experiments in Pennsylvania were successful and paved the way for further research to identify new peptide immunomodulators, which resulted in research based on the other myelin epitopes, primarily MPB_83–99_, MOG_35–55_ and PLP_139–151_ [4,5,6,7,11,12,13,14,15,16,17,18,19,20,21]. This research was quickly spread to the research community in the field, all over the world.

## 4. Development of a Worldwide MS Consortium

A multi-institutional and multidisciplinary consortium was established in 1999, by Professor John Matsoukas (from the University of Patras; currently Head of NewDrug P.C, Patras Science Park, Greece) and Professor Vasso Apostolopoulos (from the Austin Research Institute Australia/Scripps Research Institute USA); currently Pro Vice-Chancellor, Research Partnerships at Victoria University Australia). The consortium comprised over 15 top universities and research Institutions worldwide (Europe, USA, Canada and Australia), and over 50 researchers have taken part in the consortium over time. Consortium members/collaborators were included, as each had expertise in various disciplines including, chemistry, structural biology, crystallography, molecular dynamics, nuclear magnetic resonance, protein chemistry, cell biology, biochemistry, molecular biology, immunology, neuropathology, animal research, clinical research and neurology clinicians. Each team approached the MS immunotherapeutics research program, using their specialist discipline areas, which together resulted in novel findings and potential new immunotherapeutics against MS. In addition, Professor John Matsoukas (organic chemist) and his team pioneered, through rational design, cyclic constraints of myelin peptides of architectural beauty, which were evaluated for efficacy and stability by members of the consortium. In addition, his team developed novel altered peptide ligands of myelin peptides. The linear and cyclic peptides, native or as altered peptide ligands, were evaluated for stability in vitro, binding affinities to major histocompatibility complex class II, efficacy in mice and rats and to human peripheral blood mononuclear cells from patients with MS by various consortium groups [6,12,13,14,22,23,24,25,26,27,28].

## 5. Optimizing Immunotherapeutics and Vaccines against MS, Using a Novel Delivery System

Professor Vasso Apostolopoulos (immunologist and crystallographer), who had developed a novel antigen delivery system against breast and ovarian cancer [29,30,31,32,33,34,35,36,37,38,39,40,41,42,43], which were translated into human clinical trials [44,45,46,47,48], applied her insights into MS research [26,49,50,51,52,53,54]. The delivery system specifically targets dendritic cells and, when applied to myelin peptides (cyclic, linear and altered peptide ligands), was able to modulate immune responses from pro-inflammatory to anti- inflammatory, with protection and reversal of EAE in animal models and altered cytokine profile in peripheral blood mononuclear cells isolated from patients with MS [26,49,50,51,52,53,54]. Over 10 candidate immunotherapeutics have been developed and are justified for their use in phase I human clinical trials.

## 6. Awarding the Inspiration and the Pioneers of This MS Research

Dr. Elizabeth Matsoukas can justifiably be proud of what she has achieved. Her pain was translated into promising global research to fight the disease. Her dream to see novel immunotherapeutics development against the disease is very close to being materialized. At last, she has seen research due to her case flourish globally. Elizabeth was honored by the Greek Academy of Athens for her dissertation and in 2018, by His Excellency, the President of the Hellenic Democracy, Mr. Prokopis Pavlopoulos, for her initial research and for being the inspiration, the spur and the motivating power of this research. In a special ceremony on 22 September 2018, in Amaliada (province of Ilida), celebrating 20 years of Medicinal Chemistry excellence in Greece, she was awarded by the president with a DNA-inspired plague made by famous sculptor Eustathios Leontis. Standing-ovation applause for her contribution was an emotional moment. In this special ceremony, the protagonists of this MS research, Professors Apostolopoulos, Rostami and Matsoukas, were also awarded, as well Professor Harald zur Hausen, for his contribution to science and society. In addition, Professor Vasso Apostolopoulos and Nobel Laureate Professor Harald zur Hausen received an award for career excellence by His Excellency, the President of the Hellenic Democracy, Mr. Prokopis Pavlopoulos. Last year, Professor John Matsoukas and Professor Vasso Apostolopoulos, were each independently awarded the Salus Index Award, from New Times Publishing, for outstanding career achievements, including their work on MS.

## 7. Conclusions

The development of drugs, immunotherapeutics and vaccines against diseases is a long process, often taking researchers a lifetime. Researchers often work in silos, limiting their research output; as such, the breaking down of silos would improve research outcomes. Here, we provided an insight of a multi-institutional and multidisciplinary consortium which was developed over 20 years ago that has led to the identification and development of over 10 candidate immunotherapeutics against MS. Today, most research-funding bodies, require multi-institutional and multidisciplinary teams in order to be successful in grant applications. Most importantly, alliances are required to get to the target of the research.
